# Identification and validation of autophagy-related genes in SSc

**DOI:** 10.1515/med-2024-0942

**Published:** 2024-04-05

**Authors:** Chen Liu, Xiaofang Guo, Maoyun Wei, Jiaxin Xie, Xuting Zhang, Qing Qi, Ke Zhu

**Affiliations:** Department of Dermatology, Shenzhen People’s Hospital, Shenzhen, Guangdong Province, China; The First Clinical Medical College, Guangzhou University of Chinese Medicine, Guangzhou, Guangdong Province, China; Department of Dermatology, Second Hospital Affiliated to Guangzhou Medical University, Guangzhou 510260, China; Department of Dermatology, The First Affiliated Hospital, Guangzhou University of Chinese Medicine, Guangzhou, Guangdong Province, China; Department of Dermatology, Second Hospital Affiliated to Guangzhou Medical University, No. 250 Changgang Dong Road, Guangzhou 510260, China; Department of Dermatology, The First Affiliated Hospital, Guangzhou University of Chinese Medicine, Airport Road No.16 Compound, Guangzhou, Guangdong Province, China

**Keywords:** systemic sclerosis, autophagy, bioinformatics, immune infiltration

## Abstract

Multiple organs are affected by the complex autoimmune illness known as systemic sclerosis (SSc), which has a high fatality rate. Genes linked to autophagy have been linked to the aetiology of SSc. It is yet unknown, though, whether autophagy-related genes play a role in the aetiology of SSc. After using bioinformatics techniques to examine two databases (the GSE76885 and GSE95065 datasets) and autophagy-related genes, we were able to identify 12 autophagy-related differentially expressed genes that are linked to the pathophysiology of SSc. Additional examination of the receiver operating characteristic curve revealed that SFRP4 (AUC = 0.944, *P* < 0.001) and CD93 (AUC = 0.904, *P* < 0.001) might be utilized as trustworthy biomarkers for the diagnosis of SSc. The SSc group’s considerably greater CD93 and SFRP4 expression levels compared to the control group were further confirmed by qRT-PCR results. The autophagy-related genes SFRP4 and CD93 were found to be viable diagnostic indicators in this investigation. Our research sheds light on the processes by which genes linked to autophagy affect the pathophysiology of SSc.

## 1 Introduction

Systemic sclerosis (SSc) is a chronic connective tissue disorder characterized by vascular lesions, immune abnormalities and progressive fibrosis [1]. SSc has a low clinical diagnosis rate during the early stage owing to its non-specific clinical symptoms [2,3]. As the disease progresses, visceral organ involvement and fatal complications, including pulmonary hypertension, cardiac disease and renal crisis, are commonly reported [1,4]. Moreover, SSc has the highest mortality rates among the autoimmune diseases as effective treatment methods are limited [1]. Thus, early diagnosis and intervention could effectively alter the course of the disease and improve the prognosis of SSc.

Autophagy is a highly conserved process of degradation and recycling in eukaryotes and plays a critical role in the survival and maintenance of cells [5]. Autophagy dysfunction has been associated with numerous autoimmune diseases including SSc, systemic lupus erythematosus, multiple sclerosis and rheumatoid arthritis [6]. Additionally, autophagy has also been reported in various pathogenic mechanisms of SSc [7,8]. For example, autophagy is activated in patients with SSc and experimental models in a transforming growth factor-β (TGFβ)-dependent epigenetic-regulation manner [9]. Furthermore, the activation of fibroblasts in SSc skin promoted autophagy through the upregulation of PGC-1α [10]. Although a link between autophagy and SSc pathogenesis has been established, the role of autophagy-related genes in SSc remains unclear. Therefore, exploring the regulatory mechanism of autophagy-related genes in the pathogenesis of SSc is of great importance.

In this study, we analysed gene data collected from the Gene Expression Omnibus (GEO) and GeneCards databases and obtained critical autophagy-related differentially expressed genes (autophagy-related DEGs). Bioinformatics techniques, such as enrichment analysis and immune cell infiltration, explored the mechanism of autophagy-related DEGs in SSc pathogenesis. Receiver operating characteristic (ROC) curve analysis was further performed to evaluate the potential biomarker value of the 12 autophagy-related DEGs in SSc. Finally, we verified the expression levels of essential autophagy-related DEGs which might contribute to SSc in blood samples of included individuals by qRT-PCR. Thus, this study aimed to garner an in-depth analysis of the autophagy-related regulatory mechanisms and therapeutic targets for SSc.

## 2 Materials and methods

### 2.1 Data collection and pre-processing

**Figure 1:**
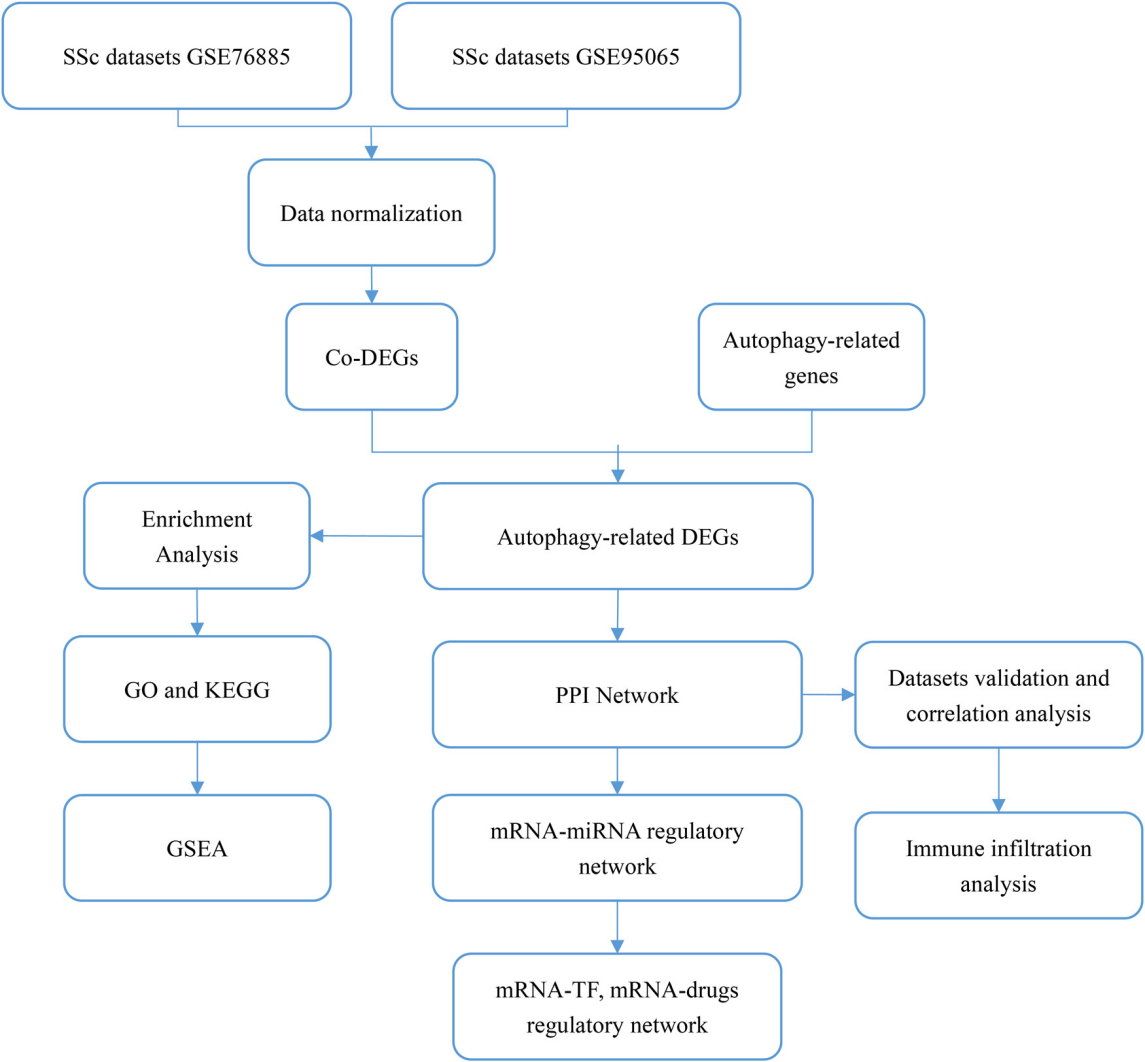
Flowchart for the identification of autophagy-related DEGs in SSc. SSc, systemic sclerosis; Co-DEGs, common differentially expressed genes; autophagy-related DEGs, autophagy-related differentially expressed genes; GO, gene ontology; KEGG, kyoto encyclopedia of genes and genomes; mRNA, messenger RNA; miRNA, microRNA; TF, transcription factor; PPI, protein–protein interaction; GSEA, gene set enrichment analysis.

The overall workflow of this study is shown in Figure 1. The gene expression profiles [11,12] (GSE76885 and GSE95065) of SSc were obtained from the GEO database (http://www.ncbi.nlm.nih.gov/geo/) via the R package GEO query [13]. From GSE76885, a total of 194 samples, including 59 patients with SSc and 18 healthy controls, were obtained. Moreover, 33 samples from the GSE95065 dataset, which included 18 patients with SSc and 15 healthy controls, were obtained. All samples of GSE95065 and a few from GSE76885 were included in this study. Then, the R package “limma” [14] was applied to remove inter-batch differences, normalize datasets and annotate probes. Autophagy-related genes were collected from the GeneCards database [15]. Furthermore, “Autophagy” was used as a keyword, identifying 7,327 genes.

### 2.2 Screening of DEGs

The samples from GSE76885 and GSE95065 were divided into the SSc and normal groups. The groups were further processed using the DESeq2 [16] package of R to obtain DEGs. DEGs with *p*.adj <0.05 and |log FC| >0.5 were considered statistically significant. Additionally, common DEGs (Co-DEGs) were identified after the intersection of DEGs in GSE76885 and GSE95065. To obtain the autophagy-related DEGs, the intersection of Co-DEGs and autophagy-related genes was performed.

### 2.3 Functional enrichment analysis

Autophagy-related genes were used for functional enrichment analysis to identify the potential mechanisms and functions. Gene ontology (GO) [17] terms and Kyoto Encyclopedia of Genes and Genomes (KEGG) [18] enrichment analysis were performed using the clusterProfiler [19] package of R. *P* < 0.05 and false discovery rate (FDR) (*q* value) <0.05 were considered statistically significant. The *P* value correction method was Benjamini–Hochberg (BH).

### 2.4 Gene set enrichment analysis (GSEA)

The genes in GSE76885 and GSE95065 were divided into two groups of high and low phenotypic correlation based on the phenotypic correlation values. Then, GSEA [20] was conducted using the clusterProfiler package in R for all the gene sets in the two groups. The parameters used in GSEA were as follows: seed, 2020; calculation number, 10,000; genes contained in each gene set, at least 10; the number of genes contained at most 500 and the correction method of the *P* value, BH. The gene set h.all.v7.2.symbols.gmt was obtained from the Molecular Signatures Database (MSigDB) [21], and the screening criteria for significant enrichment were *P* < 0.05 and FDR (*q* value) <0.05.

### 2.5 Protein–protein interaction (PPI) network analysis

Autophagy-related DEGs were used to construct a PPI network using the STRING database [22]. The minimum required interaction score of low confidence >0.150 was set as a standard reference. The interaction file including the source and target nodes was used to visualize the PPI networks using the Cytoscape software [23].

### 2.6 Construction of TF–mRNA, mRNA–miRNA and mRNA–drugs regulatory networks

Transcription factors (TF) regulate gene expression by interacting with target genes (mRNA) in the post-transcriptional stage. Based on the intersection of TFs retrieved from the ChIPBase database [24], the regulatory effects of TFs on autophagy-related DEGs were analysed. Furthermore, the intersection of miRNA related to autophagy-related DEGs was performed using the StarBase database [25] to analyse the relationship between them. To explore the interaction between autophagy-related DEGs and drugs, direct and indirect drug targets of autophagy-related DEGs were predicted using the cComparative Toxicogenomics Database [26,27]. Finally, TF–mRNA, mRNA–miRNA and mRNA–drugs regulatory networks were visualized using the Cytoscape software.

### 2.7 ROC analysis

The ROC [28] curves were drawn using the R package survival rock to identify the relationship between the survival time and survival status of patients with SSc and autophagy-related DEGs. The area under the ROC curve (AUC) was calculated, with an AUC close to 1 indicating a better diagnosis. Moreover, an AUC value between 0.7 and 0.9 indicates a certain accuracy and an AUC value above 0.9 indicates a higher accuracy.

### 2.8 Immune cell infiltration analysis using single-sample GSEA (ssGSEA)

The relative abundance of each immune cell infiltration was quantified using the ssGSEA algorithm. First, various infiltrating immune cell types were labelled, such as activated dendritic cells, activated CD8+ T cells, neutrophils, natural killer T cells, T follicular helper cells and other human immune cell subtypes. Second, the enrichment fraction calculated using ssGSEA was used to represent the relative abundance of each immune cell infiltration in each sample. Finally, the R package “ggplot2” was used to visualize the correlation between immune cells and autophagy-related DEGs.

### 2.9 SSc patients and healthy individuals

A total of three SSc patients (cases) and three healthy individuals (controls) were obtained from The First Affiliated Hospital of Guangzhou University of Chinese Medicine. Clinical characteristics of the patients with SSc are listed in Table S1. The diagnosis of SSc was followed according to the 2013 classification criteria for SSc [29] (scores ≥ 9). The three healthy individuals were recruited from the hospital’s health checkup centre. Blood samples from patients with SSc and healthy controls included in this study were collected in a 2 mL EDTA anticoagulation tube (IMPROVACUTER^®^, China). This study was conducted in accordance with the Declaration of Helsinki and approved by the Medical Ethics Committee of the hospital (No. ZYYECK[2018]107). All the participants have written the informed consent. Venous blood was collected from all cases and controls who participated in the study.

### 2.10 RNA extraction and qRT-PCR

Total RNA was extracted from blood samples of each participant using TRIzol reagent (CWBIO, Jiangsu, China) according to the manufacturer’s instructions, RNA concentrations were detected using a BIOTEK multifunctional enzyme marker (Thermo, USA). The cDNA synthesis was performed using the PrimeScript^TM^ RT Master Mix (TaKaRa, Japan). The target mRNA expression levels were normalized to ACTB internal control gene according to the instructions of PowerUP^TM^SYBR^TM^ Green Master Mix (Thermo, USA). The 2^−△△CT^ method was used for data analysis. All primer sequences of the genes and ACTB are listed in Table S2.


**Ethics approval and consent to participate:** The studies involving human participants were reviewed and approved by the Ethics Committee of The First Affiliated Hospital, Guangzhou University of Chinese Medicine (K-2023-091). The participants provided their written informed consent to participate in this study.

## 3 Results

### 3.1 Analysis of DEGs associated with SSc

**Figure 2:**
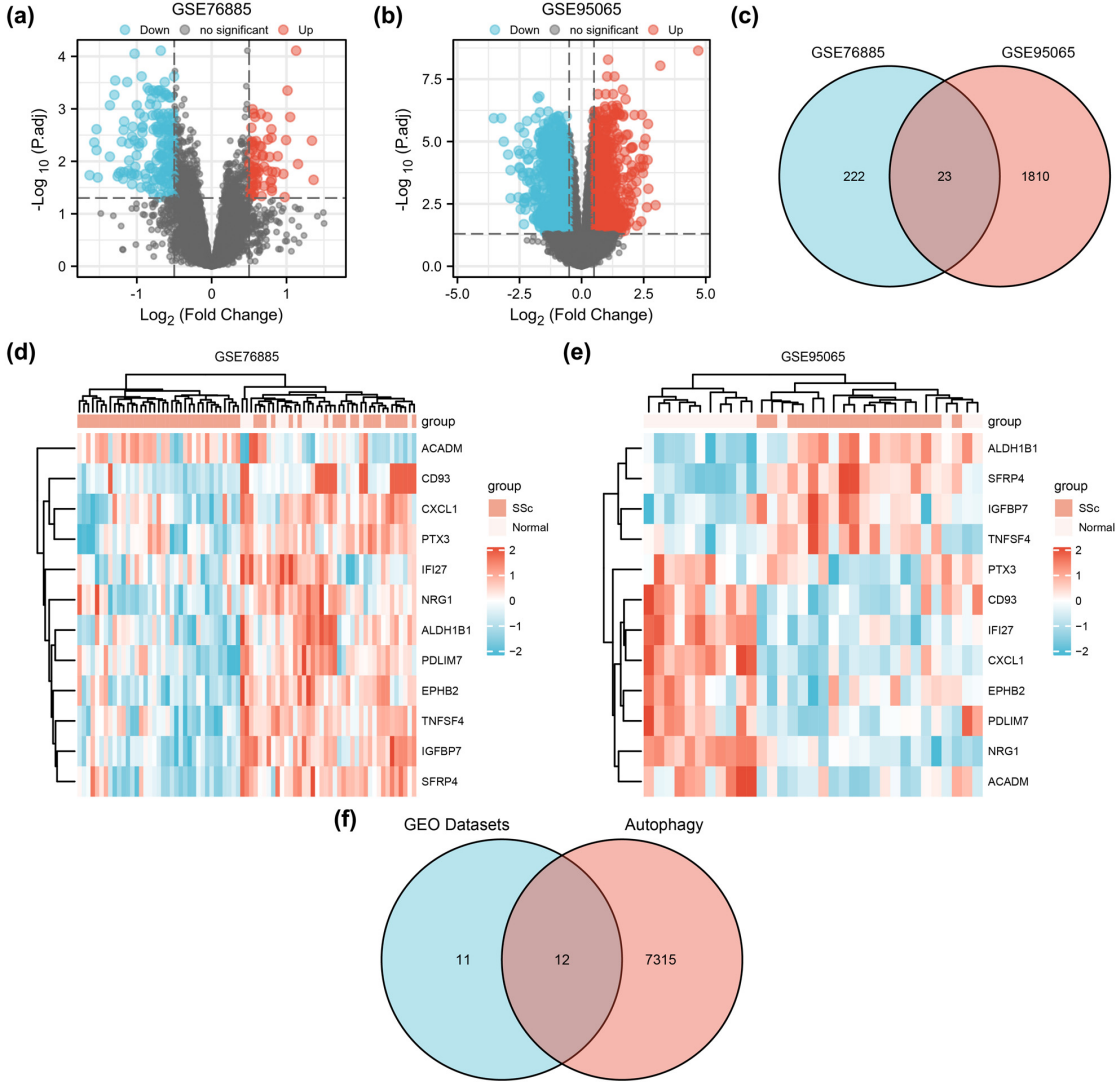
SSc differential gene expression analysis. (a) and (b) The volcano plot illustrates DEGs between normal and SSc after analysis of the (a) GSE76885 and (b) GSE95065 datasets with DESeq2. (c) Venn diagram of Co-DEGs intersection between DEGs from GSE76885 and GSE95065 datasets. (d) and (e) Heatmap of Co-DEGs in GSE76885 and GSE95065, respectively. (f) Venn diagram shows the intersection between CoDEGs and autophagy-related genes. DEGs: differentially expressed genes; Co-DEGs, common expression of differentially expressed genes.

The GSE76885 dataset contained 245 DEGs, wherein 69 were upregulated and 69 were downregulated (Figure 2a). Of the 1,833 DEGs in GSE95065, 872 were upregulated and 961 were downregulated (Figure 2b). Co-DEGs identified from the two datasets were filtered in the Venn diagram (Figure 2c), and the heatmaps were used for visualization (Figure 2d and e). Finally, a total of 12 autophagy-related DEGs were identified (Figure 2f), including *EPHB2, IFI27, ALDH1B1, PDLIM7, IGFBP7, SFRP4, NRG1, CXCL1, CD93, TNFSF4, PTX3* and *ACADM*.

**Figure 3:**
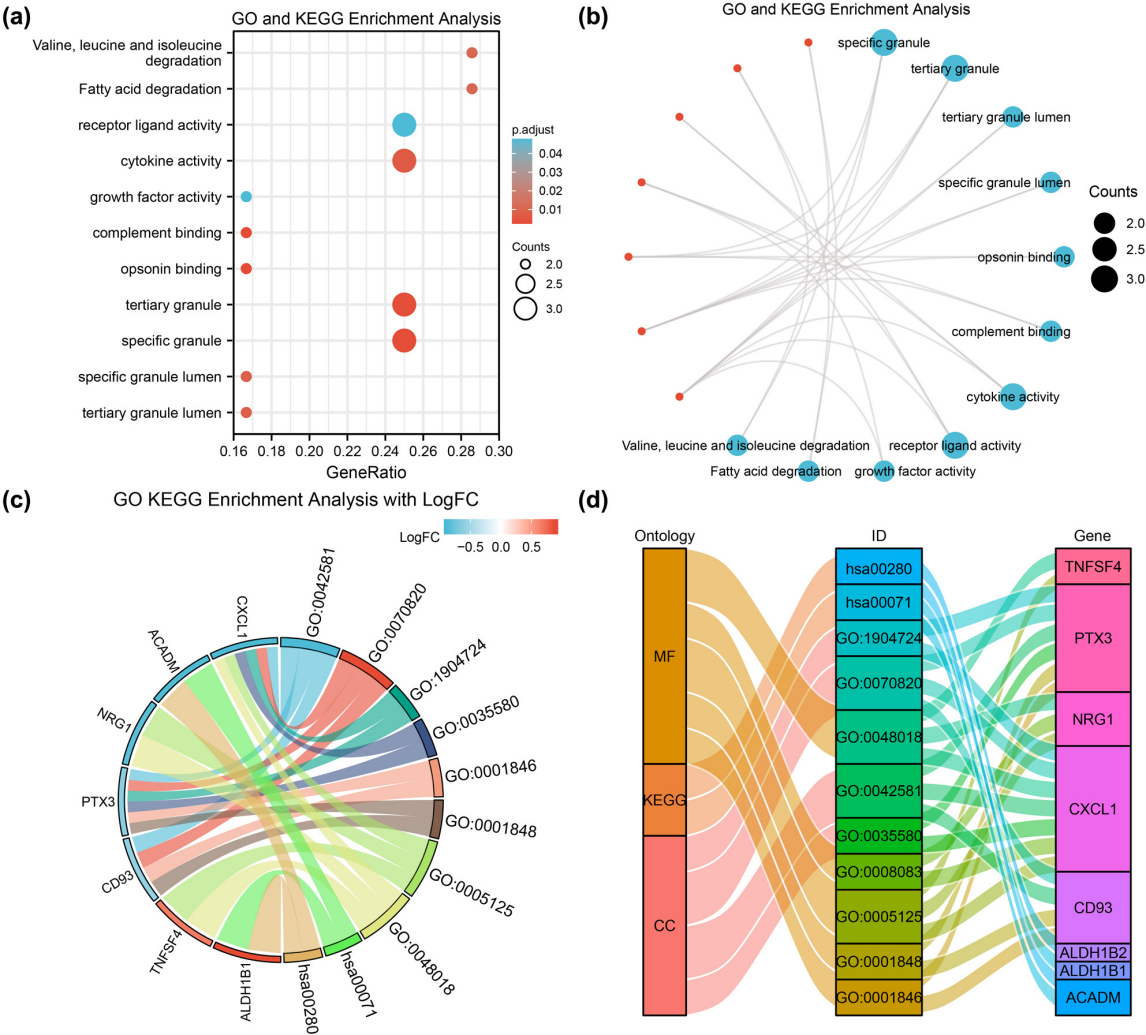
GO and KEGG enrichment analysis for autophagy-related DEGs. (a)–(d) GO and KEGG enrichment shown in (a) Bubble plots, (b) network diagram, (c) joint log FC chord diagram and (d) Sankey diagram. GO, gene ontology; KEGG, Kyoto Encyclopedia of Genes and Genomes; MF, molecular function; CC, cellular component.

GO and KEGG analysis were applied to explore the potential biological functions of the 12 autophagy-related DEGs (Tables S3 and S4). GO analysis indicated that the 12 autophagy-related DEGs were mainly enriched in the cellular components of the specific granules, tertiary granules, tertiary granule lumen and specific granule lumen. The molecular functions of the 12 autophagy-related DEGs were mainly related to opsonin binding, complement binding, cytokine activity, receptor–ligand activity and growth factor activity (Figure 3a). Furthermore, KEGG pathway analysis revealed that the 12 autophagy-related DEGs were strongly associated with valine, leucine and isoleucine degradation pathways and fatty acid degradation. The GO and KEGG analyses are presented using network diagrams, chordal graphs and Sankey diagrams (Figure 3b–d).

**Figure 4:**
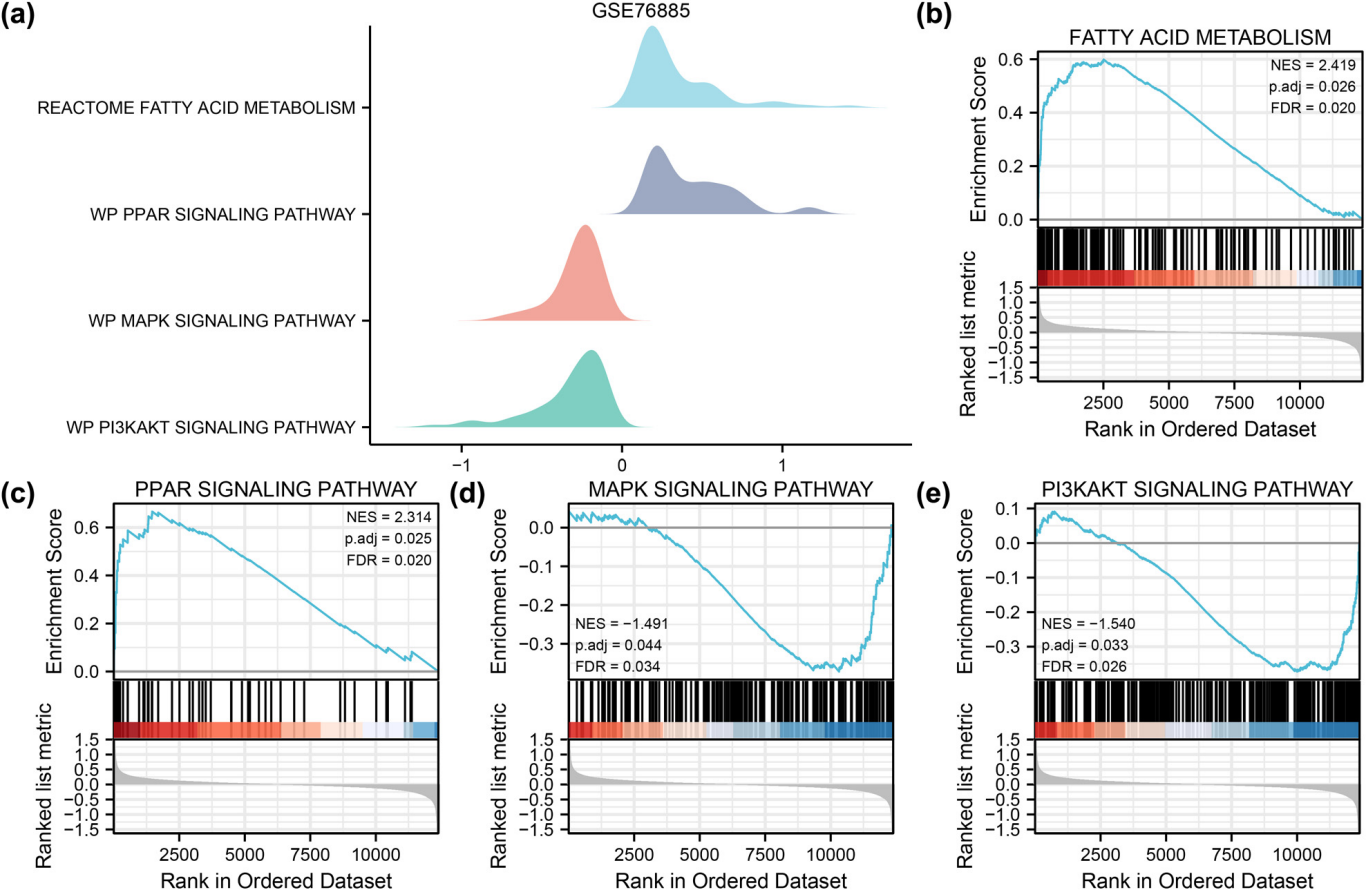
GSEA for SSc dataset GSE76885. (a) Mountain diagrams show four types of enriched biological functions. (b)–(d) GSEA used to validate the fatty acid metabolism (b), PPAR signalling pathway (c), MAPK signalling pathway (d) and PI3K/AKT signalling pathway (e). GSEA: gene set enrichment analysis; SSc: systemic sclerosis.

**Figure 5:**
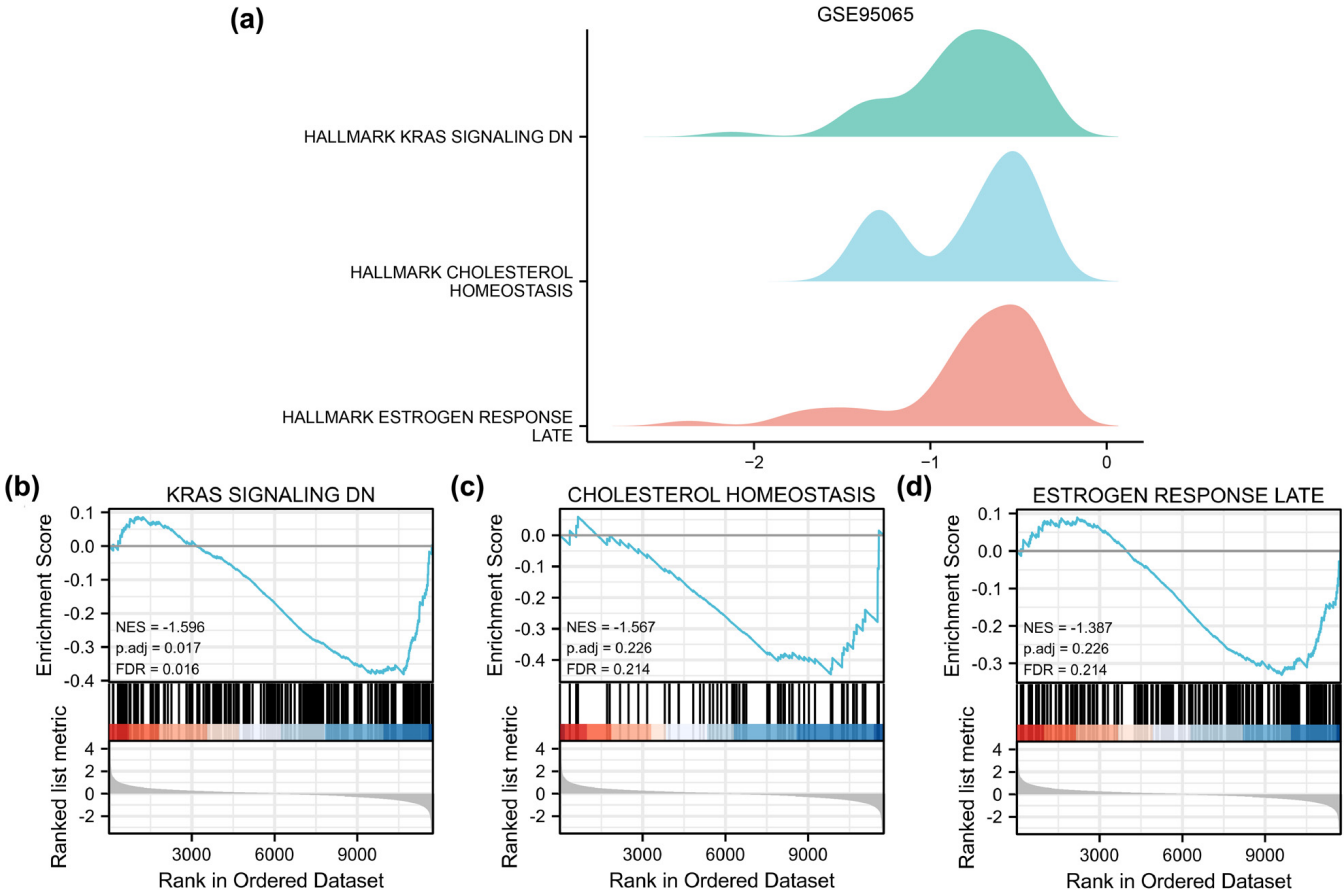
GSEA for SSc dataset GSE95065. (a) Mountain diagrams show three types of enriched biological functions. (b)–(d) GSEA used to validate the KRAS signalling DN (b), cholesterol homeostasis (c) and estrogen response late (d). GSEA: gene set enrichment analysis; SSc: systemic sclerosis.

GSEA of the DEGs in the GSE76885 and GSE95065 datasets evaluated the potential biological functions and involved pathways (Tables S5 and S6). The DEGs of GSE76885 significantly affected fatty acid metabolism, the PPAR signalling pathway, the MAPK signalling pathway and the PI3K/AKT signalling pathway (Figure 4). Additionally, the DEGs of GSE95065 significantly affected KRAS signalling, cholesterol homeostasis, estrogen response, and several related biological functions (Figure 5). These findings indicate that various metabolism-related pathways are involved in the development of SSc.

### 3.2 Construction of the PPI interaction network and TF–mRNA, mRNA–miRNA and mRNA–drugs regulatory networks

**Figure 6:**
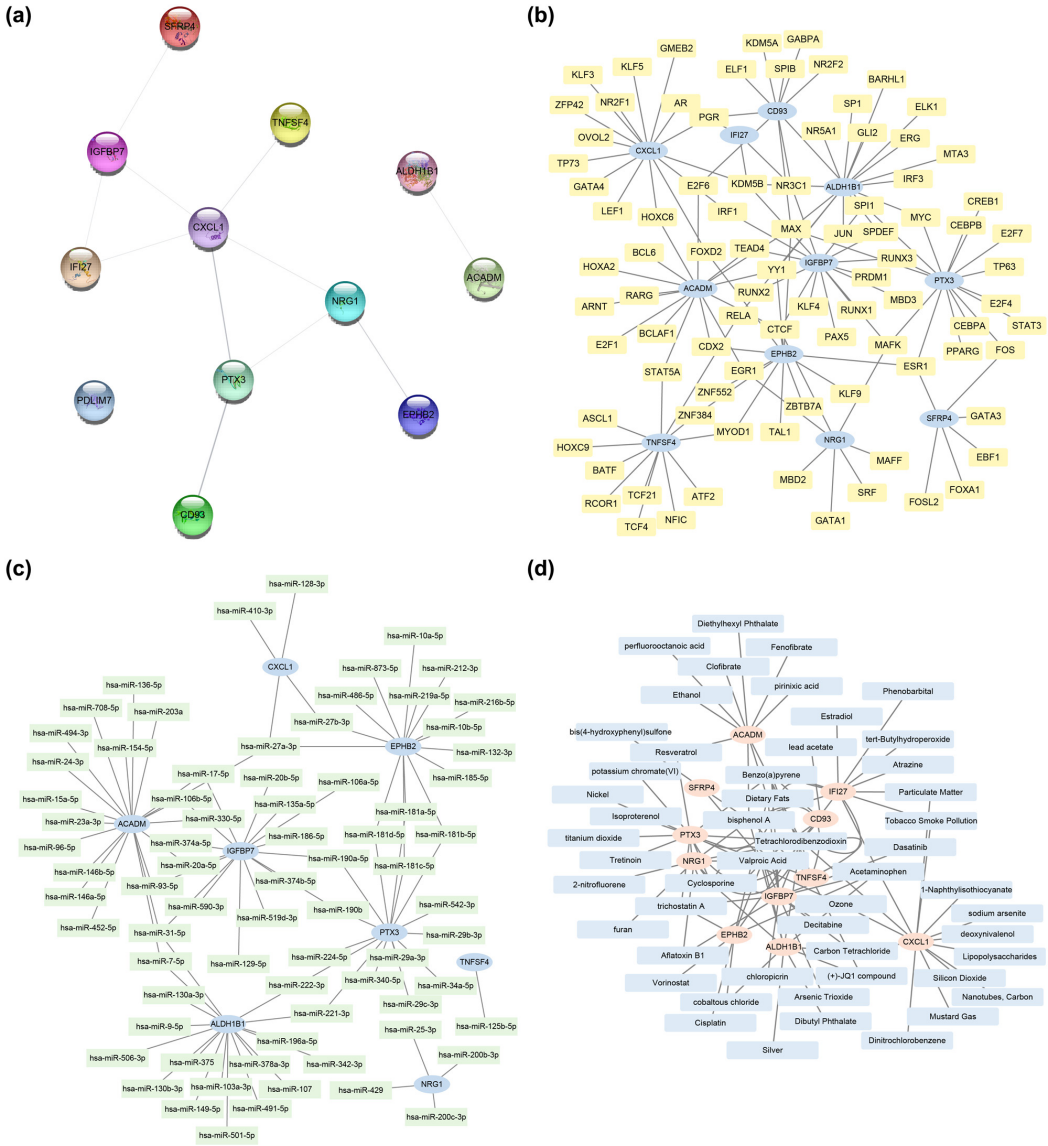
Regulatory network of autophagy-related DEGs. (a)–(d) The PPI network (a) and the regulatory network of mRNA–TF (b), mRNA–miRNA (c), mRNA drugs (d) of autophagy-related DEGs. PPI: protein–protein interaction network.

A PPI network of the 12 autophagy-related DEGs was established to explore their relationship with each other (Figure 6a). Of these, 11 autophagy-related DEGs were related to each other: *EPHB2, IFI27, ALDH1B1, IGFBP7, SFRP4, NRG1, CXCL1, CD93, TNFSF4, PTX3* and *ACADM*. Following this, the mRNA–TF regulatory network was constructed, wherein 11 mRNAs of autophagy-related DEGs and 126 TFs were identified (Figure 6b). Then, the mRNA–miRNA regulatory network was constructed (Figure 6c). Among them, eight mRNAs (*EPHB2, ALDH1B1, IGFBP7, NRG1, CXCL1, TNFSF4, PTX3* and *ACADM*) and 93 miRNAs were included. Finally, the mRNA–drugs regulatory network was constructed and visualized, in which the same 11 mRNAs along with 106 drugs or molecular compounds were screened (Figure 6d).

### 3.3 Dataset validation and ROC analysis

**Figure 7:**
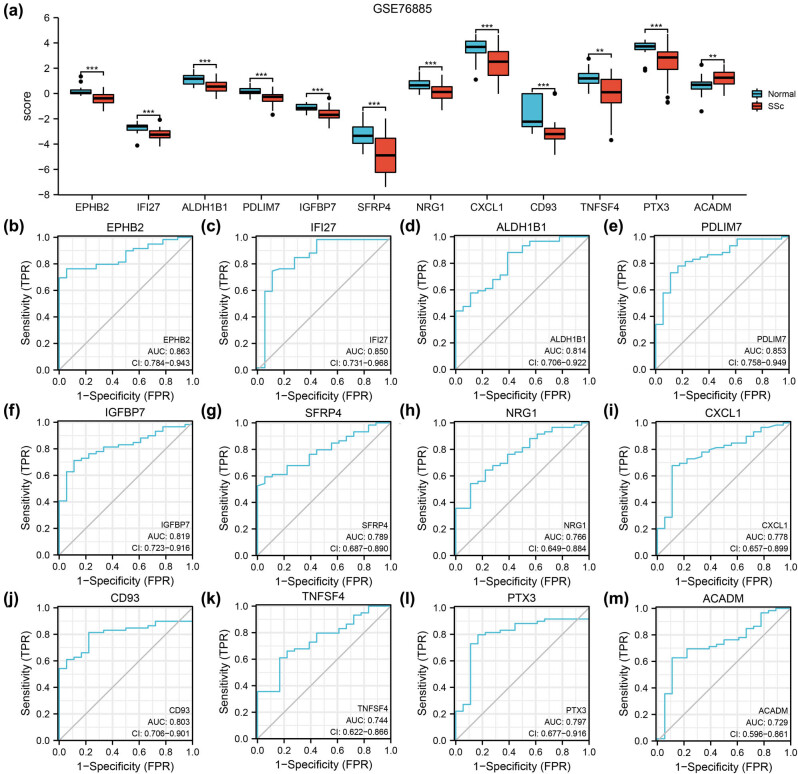
Validation of datasets GSE76885 and ROC curve analysis. The expression and diagnostic performance of the 12 genes in GSE76885. (a) Autophagy-related DEGs expression difference between normal and SSc groups. (b)–(m) ROC curves in dataset GSE76885 of autophagy-related DEGs EPHB2 (b), IFI27 (c), ALDH1B1 (d), PDLIM7 (e), BP7 (f), SFRP4 (g), NRG1 (h), CXCL1 (i), CD93 (j), TNFSF4 (k), PTX3 (l) and ACADM (m). ROC: receiver operator characteristic curve; AUC: area under the curve; SSc: systemic sclerosis. ** represents *P* value <0.01; *** represents *P* value <0.001.

**Figure 8:**
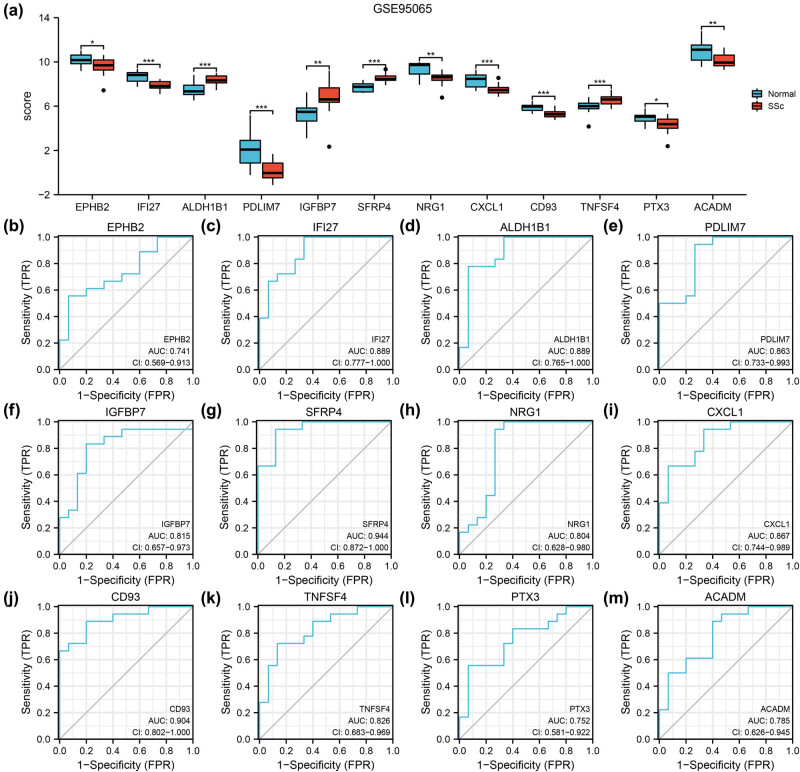
Validation of datasets GSE95065 and ROC curve analysis. The expression and diagnostic performance of the 12 genes in GSE95065. (a) Autophagy-related DEGs expression difference between normal and SSc groups. (b)–(m) ROC curves in dataset GSE76885 of autophagy-related DEGs EPHB2 (b), IFI27 (c), ALDH1B1 (d), PDLIM7 (e), IGFBP7 (f), SFRP4 (g), NRG1 (h), CXCL1 (i), CD93 (j), TNFSF4 (k), PTX3 (l) and ACADM (m). ROC: receiver operator characteristic curve; AUC: area under the curve; SSc: systemic sclerosis. * *P* < 0.05; ** *P* < 0.01; *** *P* < 0.001.

To further verify the expression differences of the 12 autophagy-related DEGs in the GSE76885 and GSE95065 datasets, comparison graphs of the SSc and normal groups were drawn (Figures 7a and 8a). All autophagy-related DEGs were statistically significant (*P* < 0.05) in the two datasets. Notably, CD93 and SFRP4 were statistically significant (*P* < 0.001). The diagnostic values of autophagy-related DEGs were determined using ROC curve analysis. The 12 genes were identified in GSE76885 and GSE95065 and exhibited an AUC > 0.7 (Figures 7 and 8), which indicated that they have a significant correlation with the diagnosis of SSc. Furthermore, the AUCs for *SFRP4* and *CD93* in GSE95065 were 0.944 and 0.904, respectively (Figure 7g and j).

### 3.4 Immune infiltration analysis

**Figure 9:**
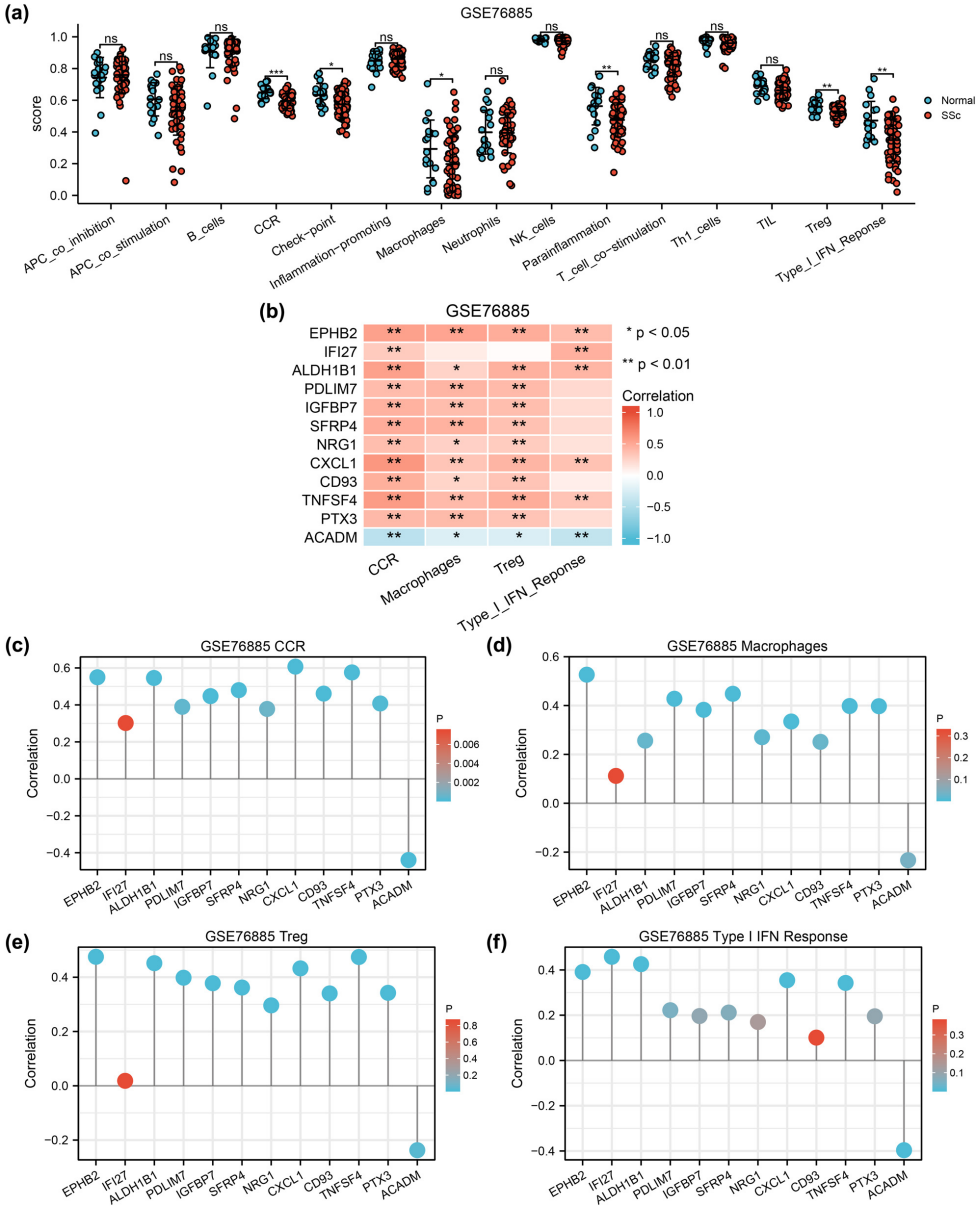
Immune infiltration analysis of datasets GSE76885 by ssGSEA algorithm. (a) Comparison graph of the proportion 29 types of immune cells between normal and SSc groups in GSE76885. ns *P* ≥ 0.05; * *P* < 0.05; ** *P* < 0.01; *** *P* < 0.001. (b) Heat map of the correlation between four infiltrated immune cells and autophagy-related DEGs. (c)–(f) Lollipop diagram of the correlation between autophagy-related DEGs and abundance of four immune cell infiltration, CCR (c), macrophages (d), Treg (e) and Type I IFN response (f). ssGSEA: single sample gene set enrichment analysis; SSc: systemic sclerosis.

**Figure 10:**
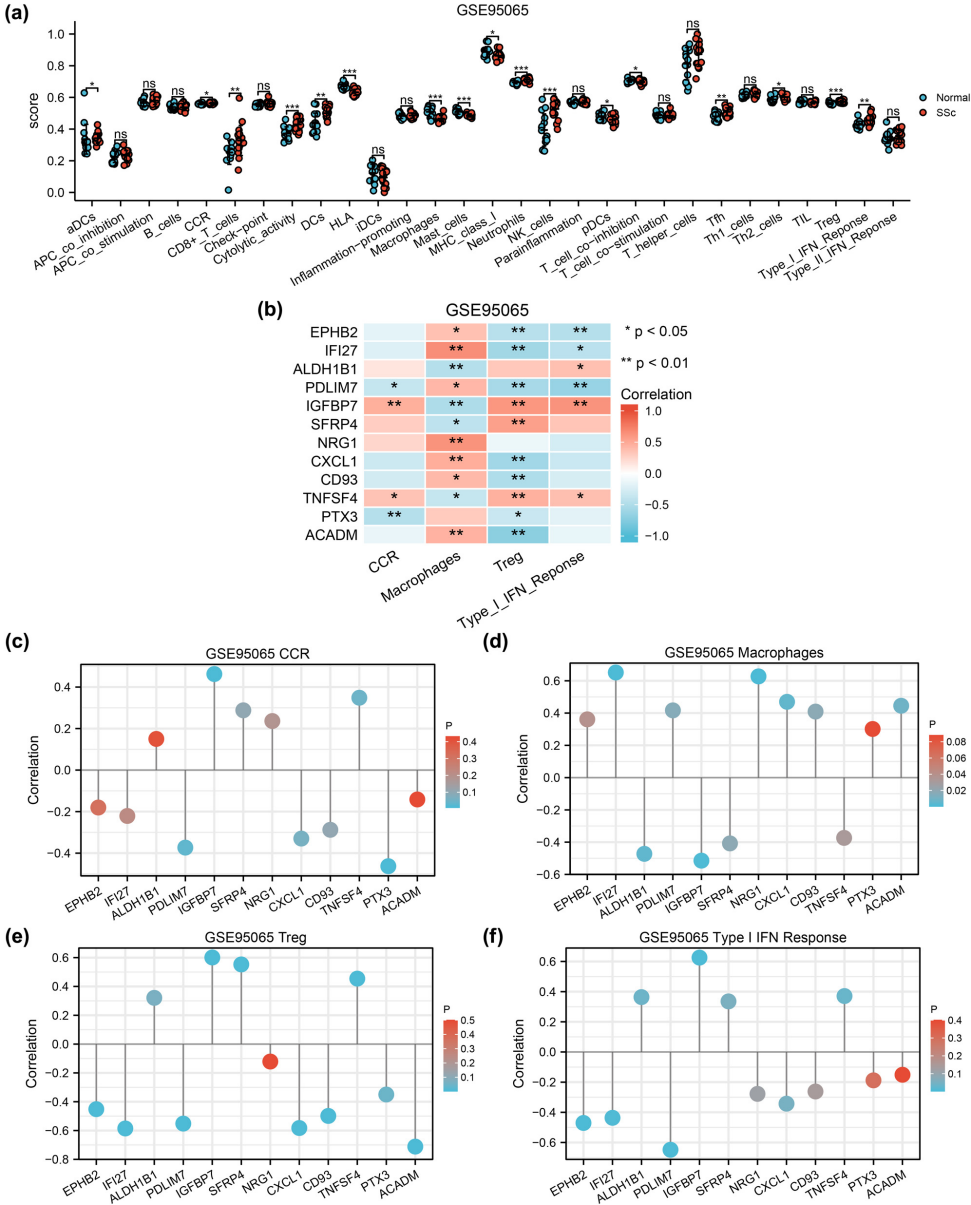
Immune infiltration analysis of datasets GSE95065 by ssGSEA algorithm. (a) Comparison graph of the proportion 29 types of immune cells between normal and SSc groups in GSE95065. ns *P* ≥ 0.05. (b) Heat map of the correlation between four infiltrated immune cells and autophagy-related DEGs. (c)–(f) Lollipop diagram of correlation between autophagy-related DEGs and abundance of four immune cell infiltration, CCR (c), macrophages (d), Treg (e) and Type I IFN response (f). ssGSEA: single sample gene set enrichment analysis; SSc: systemic sclerosis; * *P* < 0.05; ** *P* < 0.01; *** *P* < 0.001.

Immune infiltration analysis revealed the correlation between the 29 immune cells in GSE76885 or GSE95065 using group comparison maps (Figures 9a and 10a). CCR, macrophages, Treg and Type I IFN response cells showed statistically significant differences (*P* < 0.05). The immune infiltration analysis of these four immune cells was identified using correlation heat maps (Figures 9b and 10b). Finally, the lollipop charts were used to visualize the correlation between the four infiltrated immune cells and the 12 autophagy-related DEGs in the GSE76885 (Figure 9c–f) and GSE95065 (Figure 10c–f) datasets.

### 3.5 Validation the differentially expressed autophagy-related genes in SSc patients

**Figure 11:**
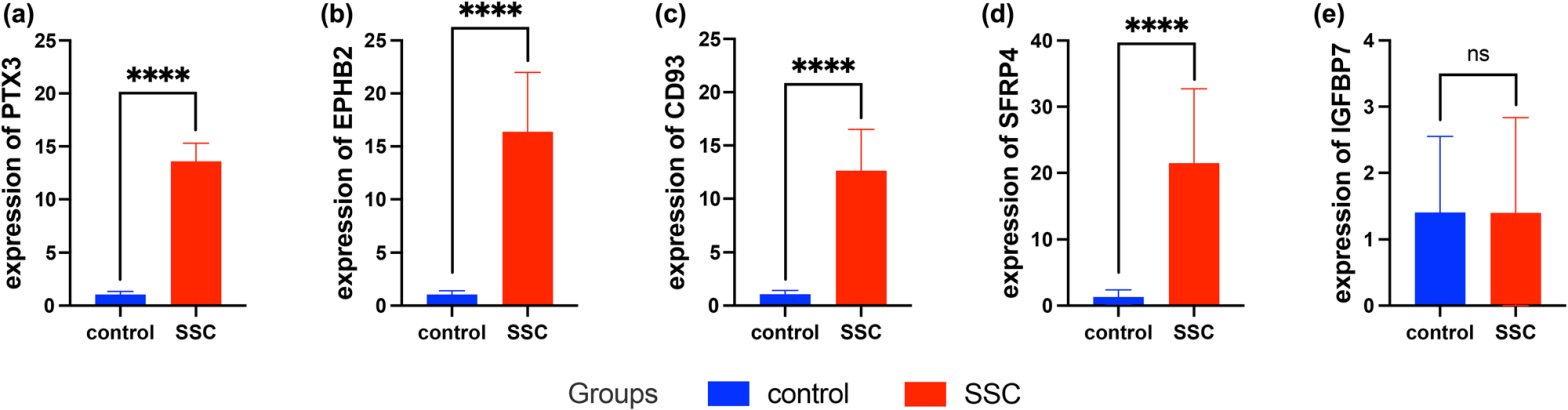
mRNA expression levels of five autophagy-related DEGs were measured in SSc and normal samples. (a)–(e) Expression level of (a) PTX3, (b) EBHP2, (c) CD93, (d) SFRP4 and (e) IGFBP7. ***P < 0.001. Abbreviation: autophagy-related DEGs, autophagy-related differentially expressed genes; ns, non-significant.

To further increase our confidence in the analysis results, the expression levels of several autophagy-related DEGs were further identified by qRT-PCR in different sample groups. Compared with the normal control, the expression levels of CD93, PTX3, EBHP2 and SFRP4 were significantly up-regulated in SSc blood samples (Figure 11a–d). However, the expression levels of IGFBP7 showed no significant difference between the two groups (Figure 11e). IFI27, ALDH1B1, PDLIM7, NRG1, CXCL1, TNFSF4 and ACADM were not detected by qRT-PCR.

## 4 Discussion

Despite being an orphan illness with a low frequency, SSc causes disproportional morbidity and mortality, which is exacerbated by delays in diagnosis due to non-specific early symptoms and the average clinician’s inexperience in its detection and therapy [30]. Autophagy is a pivotal recycling and degradative system in eukaryotic cells; identifying the roles of autophagy-related genes or proteins in disease has become a research hotspot. Recent studies has indicated that autophagy is critical for the development of SSc [31]. Previous research has found that skin specimens from SSc patients have higher levels of LC3 immunoreactivity than healthy controls, while another study found that SSc fibroblasts have defective autophagy [32,33]. However, studies investigating the regulatory mechanism of autophagy-related genes in SSc are scarce. Autophagy dysregulation may play a role in the pathophysiology and clinical course of SSc, and aberrantly produced autophagy proteins may serve as potential therapeutic targets. Therefore, this study aims to find key autophagy-related genes in SSc and identify their potential regulated mechanisms.

In this study, we identified 12 autophagy-related DEGs, from the Co-DEGs of two gene expression profiles and autophagy-related genes. A series of bioinformatics analyses and ROC curve analyses were applied to confirm the regulatory mechanisms and diagnostic significance of these genes in SSc. Among the 12 autophagy-related DEGs identified, all of them had an AUC ＞0.7, with *CD93* and *SFRP4* exhibiting high sensitivity and specificity in SSc diagnosis (*P* < 0.001, AUC = 0.904; *P* < 0.001, AUC = 0.944), respectively.

CD93, a highly glycosylated transmembrane glycoprotein, is strongly associated with inflammation and angiogenesis [34]. Although relevant studies regarding CD93 regulating angiogenesis in SSc are scarce, a recent study showed that the serum soluble CD93 (sCD93) levels in patients with SSc having a disease duration ＜6 years were significantly higher [35]. The higher the serum sCD93 levels, the more severe the skin involvement and the shorter the duration of SSc. Importantly, there is an aberrant immune response persistent in SSc. In the early phases of SSc, serum C-reactive proteinlevels are highly elevated along with interleukin 6 (IL-6) levels [36]. Jeon et al. showed that sCD93 enhances TLR-stimulated IL-6 production when monocyte differentiation is induced [37]. Therefore, we speculated that CD93 could be essential both in angiogenesis and the inflammation progression of SSc.

SFRP4, secreted frizzled-related protein 4, is also known as secreted Wnt inhibitor [38,39]. Bayle et al. demonstrated that in Tsk mouse, an SSc animal model, SFRP4 might represent a counter-regulatory mechanism of Wnt2 and thus contributes to skin fibrosis via Tsk-Fbn matrix remodelling [40]. SFRP4 also participates in epithelial–mesenchymal transition (EMT), which is also an important event that contributes to fibrosis in SSc. SFRP4 have been demonstrated to be elevated during TGF-β-induced EMT *in vitro* [41]. Additionally, serum levels of SFRP4 are also closely related to pulmonary and cutaneous fibrosis severity in patients with SSc and could function as a biomarker [41]. Notably, autophagy is a key feature during EMT [42]. However, the relationship among SFRP4, autophagy, EMT and the progression of SSc remains to be investigated.

In addition, results of qRT-PCT showed that the expression levels of CD93 and SFRP4 were markedly up-regulated in SSc patients with statistical significance. We suggest that CD93 and SFRP4 may play a pivotal role in the developing of SSc and may serve as biomarkers for diagnosis, which may be beneficial in controlling autophagy levels and making appropriate diagnosis and treatment decisions for patients. Our findings may provide a foundation for future investigations into how CD93 and SFRP4 control autophagy in SSc; still, additional testing is necessary to validate our hypothesis.

Furthermore, our findings revealed that key autophagy pathways, including PI3K/AKT and MAPK signalling, were identified to play a regulatory role in the mechanism of SSc. Autophagy-related DEGs were also enriched in metabolism-related pathways, including fatty acid metabolism, cholesterol homeostasis and estrogen response late. Metabolism dysregulation could influence inflammation and fibrosis, ultimately affecting SSc progression [43]. Autophagy is primary in maintaining cellular energy homeostasis within the whole organism. Thus, the activation of autophagy could be a beneficial way to treat metabolic diseases [44]. Sirolimus, which induces autophagy by inhibiting mTORC1, has reduced the risk of atherosclerotic plaques in model animals on a high-fat or cholesterol diet compared to control groups [45]. Therefore, developing medicines targeting autophagy-metabolism could be a practical method for preventing and treating SSc.

ssGSEA identified a remarkably increased infiltration of CCR, macrophages, Treg and Type I IFN response in both the GSE76885 and GSE95065 datasets (*P* ＜ 0.05, correlation coefficient ＞0). Consistently, GO analysis also indicated that autophagy-related DEGs were involved in immune regulation. CD93 and SFRP4 expression were both positively correlated with macrophages and Treg cells. Macrophages are the main regulators of fibrosis and inflammation in SSc, with M1 macrophages producing pro-inflammatory cytokines that contribute to the inflammation. Furthermore, macrophages polarize into M2 macrophages and activate fibrosis by producing anti-inflammatory cytokines, growth factors, and chemokines including TGF-β [46]. Autophagy could regulate macrophage polarization and can be a potential therapeutic target for organ fibrosis [47]. A study demonstrated that macrophages in late wound phagocytize SFRP4 in order to persistent fibrotic signalling activity [48]. Treg is a subset of T lymphocytes; however, its role in fibrosis is controversial. The activation state of Treg cells is dynamically programmed to respond differently to different environments and immune conditions [49]. Thus, Treg cells exhibit either pro-fibrotic or anti-fibrotic effects depending on the environment. Wei et al. revealed that autophagy is crucial in protecting the lineage and survival of Treg cells, which prevents compromising their functional integrity in activating contexts [50].

The study has many drawbacks. First, due to the relatively low clinical prevalence of SSc, the number of clinical samples in our study is limited, and we need to confirm our findings in a larger SSc cohort. Second, it is also limited in the number of datasets included. Third, bioinformatics analysis used to analyse the functions of autophagy-related DEGs in the present study was limited. Furthermore, multi-omics studies and *in vivo* experiments on autophagy-related DEGs are required to comprehensively understand their functions and verify their feasibility as biomarkers. Increased study with experimental animals may improve the validity of our findings, which will be the focus of future research.

## 5 Conclusions

We identified 12 autophagy-related DEGs which were closely related to the pathogenesis of SSc using bioinformatics methods. They might contribute to SSc through autophagy by regulating metabolism and immune cell infiltration. Among these genes, CD93 and SFRP4 had the highest AUC values and were significantly up-regulated in blood samples of SSc, which might be key biomarkers for diagnosis and potential therapeutic targets. Thus, this study expands on the current knowledge of autophagy-related genes in the pathogenesis of SSc, which will help better understand their regulatory mechanisms in SSc pathogenesis and develop new diagnostic markers.

## Supplementary Material

supplementary material
