# Supplementary material for: Identification and validation of autophagy-related genes in SSc
Source: Open Med (Wars). 2024 Apr 5;19(1):20240942. doi: 10.1515/med-2024-0942 (PMC10998681; doi:10.1515/med-2024-0942)
Supplement: supplementary material [file med-2024-0942-sm.pdf]

Supplementary Materials

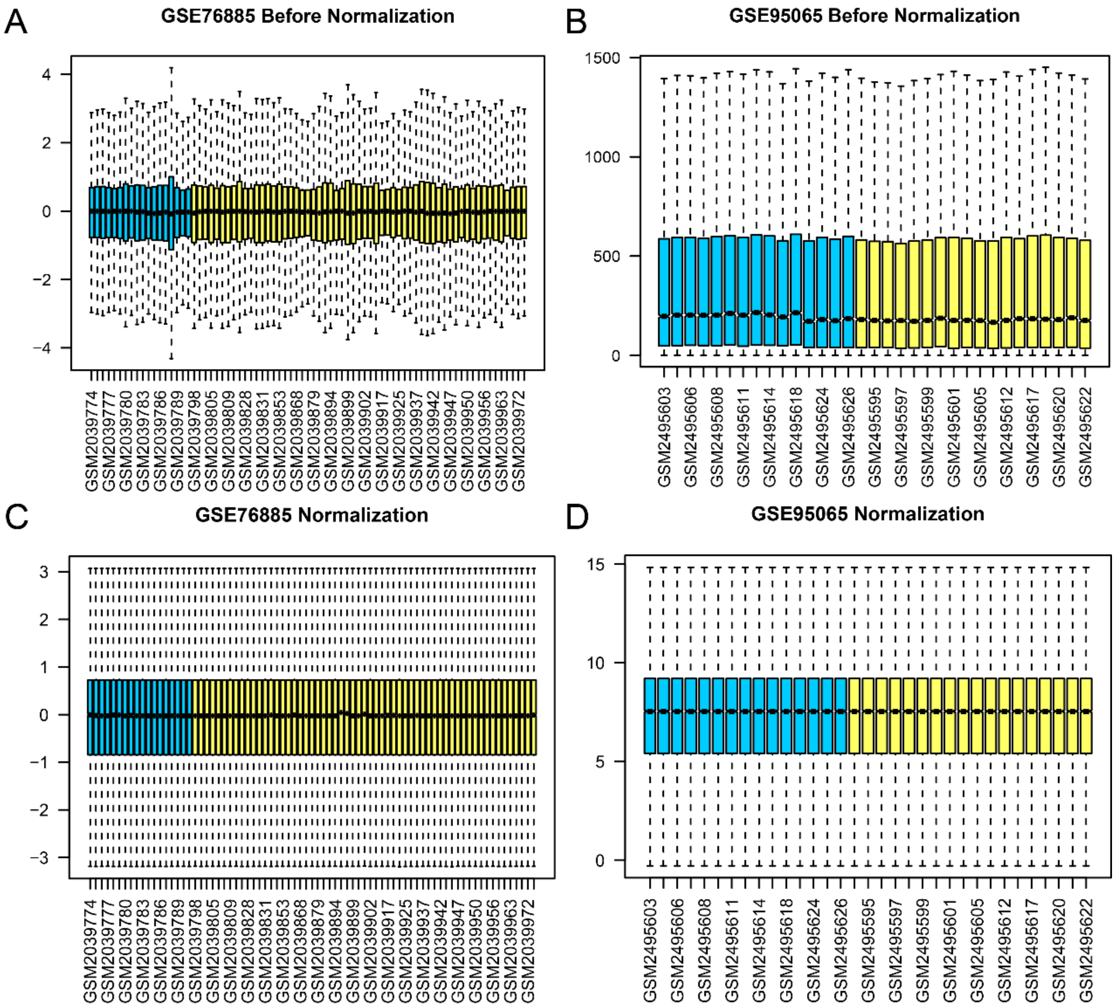

Supplementary Figure 1 . Datasets normalization. A-D Genes expression distribution of (A) GSE76885 before normalization, (B) GSE95065 before normalization; (C) GSE76885 after normalization, and (D)GSE95065 after normalization. Blue represents the control group (Normal), yellow represents the Systemic Sclerosis group (SSc)

Table S1. The clinical features of involved individuals

	Gender	Age
Case 1	female	69
Case 2	female	53
Case 3	male	27
Control 1	female	25
Control 2	male	23
Control 3	male	28

Table S2. The primers sequence

Gene	Forward Sequence	Reverse Sequence
PTX3	5′-CTTGTGGGTAAATGGTGAACT-3′	5′-CCAGATATTGAAGCCTGTGAG-3′
EBHB2	5′-GTGTGCAACGTGTTTGAGTCA-3′	5′-ACGCACCGAAAACTTCATCTC-3′
SFRP4	5′-CACACCAGACATGATGGTACAG-3′	5′-GCTGAGATACGTTGCCAAAGTT-3′
CD93	5′-CCGGAAGTAACATTGAGGGCT-3′	5′-TCTGAGTCTCGTCCTTGTCAC-3′
IGFBP7	5′- CACTGGTGCCCAGGTGTACT-3′	5′-TTGGATGCATGGCACTCATAT-3′
ACTB	5′-ATTGCCGACAGGATGCAGA-3′	5′-GAGTACTTGCGCTCAGGAGGA-3′

Table S3. Results of GO Enrichment Analysis

ONTOLOGY	ID	Description	GeneRatio	BgRatio	p-value	p.adjust	q value
CC	GO:0042581	specific granule	3/12	160/19717	1.09e-04	0.003	0.002
CC	GO:0070820	tertiary granule	3/12	164/19717	1.18e-04	0.003	0.002
CC	GO:1904724	tertiary granule lumen	2/12	55/19717	4.95e-04	0.009	0.006
CC	GO:0035580	specific granule lumen	2/12	62/19717	6.29e-04	0.009	0.006
MF	GO:0001846	opsonin binding	2/12	15/17697	4.40e-05	0.002	8.79e-04
MF	GO:0001848	complement binding	2/12	21/17697	8.79e-05	0.002	8.79e-04
MF	GO:0005125	cytokine activity	3/12	220/17697	3.84e-04	0.007	0.003
MF	GO:0048018	receptor ligand activity	3/12	482/17697	0.004	0.048	0.018
MF	GO:0008083	growth factor activity	2/12	163/17697	0.005	0.048	0.018

GO: Gene Ontology

Table S4. Result of KEGG Enrichment Analysis

ONTOLOGY	ID	Description	GeneRatio	BgRatio	p-value	p.adjust	q value
KEGG	hsa00071	Fatty acid degradation	2/7	44/8076	5.99e-04	0.011	0.007
KEGG	hsa00280	Valine， leucine and isoleucine degradation	2/7	48/8076	7.13e-04	0.011	0.007

KEGG: Kyoto Encyclopedia of Genes and Genomes

Table S5. Results of GSE76885 GSEA

Description	setSize	Enrichment Score	NES	p-value	p.adjust	q value
REACTOME_FATTY_ACID_METABOLISM	138	0.59890	2.41892	0.00238	0.02576	0.02028
WP_PPAR_SIGNALING_PATHWAY	51	0.66654	2.31393	0.00222	0.02543	0.02002
WP_CHOLESTEROL_METABOLISM_INCLUDES_BOTH_BLOCH_AND_KANDUTSCHRUSSELL_PATHWAYS	42	0.69302	2.27446	0.00230	0.02543	0.02002
KEGG_PPAR_SIGNALING_PATHWAY	53	0.64459	2.23985	0.00228	0.02543	0.02002
WP_TRIACYLGLYCERIDE_SYNTHESIS	16	0.83528	2.19918	0.00212	0.02543	0.02002
WP_OMEGA9_FA_SYNTHESIS	13	0.85400	2.14243	0.00204	0.02543	0.02002
KEGG_TERPENOID_BACKBONE_BIOSYNTHESIS	13	0.85343	2.14100	0.00204	0.02543	0.02002
REACTOME_TRIGLYCERIDE_METABOLISM	25	0.72223	2.10449	0.00224	0.02543	0.02002
REACTOME_CHOLESTEROL_BIOSYNTHESIS	22	0.73251	2.05915	0.00222	0.02543	0.02002
WP_OMEGA3OMEGA6_FA_SYNTHESIS	12	0.84563	2.05742	0.00207	0.02543	0.02002
KEGG_BIOSYNTHESIS_OF_UNSATURATED_FATTY_ACIDS	18	0.75458	2.04944	0.00210	0.02543	0.02002
REACTOME_ACTIVATION_OF_GENE_EXPRESSION_BY_SREBF_SREBP_	37	0.64209	2.03969	0.00232	0.02543	0.02002
REACTOME_ALPHA_LINOLENIC_OMEGA3_AND_LINOLEIC_OMEGA6_ACID_METABOLISM	12	0.82680	2.01163	0.00207	0.02543	0.02002
WP_CHOLESTEROL_BIOSYNTHESIS_PATHWAY	14	0.78996	2.00795	0.00211	0.02543	0.02002
KEGG_PEROXISOME	65	0.55628	2.00539	0.00229	0.02543	0.02002
REACTOME_PEROXISOMAL_LIPID_METABOLISM	25	0.68543	1.99724	0.00224	0.02543	0.02002
WP_FATTY_ACID_BIOSYNTHESIS	19	0.72484	1.99620	0.00221	0.02543	0.02002
WP_MAPK_SIGNALING_PATHWAY	188	-0.37186	-1.49096	0.00493	0.04370	0.03440
KEGG_MAPK_SIGNALING_PATHWAY	201	-0.37891	-1.52555	0.00497	0.04388	0.03455
WP_PI3KAKT_SIGNALING_PATHWAY	250	-0.37214	-1.54015	0.00316	0.03349	0.02637

GSEA: Gene Set Enrichment Analysis

Table S6. Results of GSE95065 GSEA

Description	setSize	Enrichment Score	NES	p-value	p.adjust	q value
HALLMARK_KRAS_SIGNALING_DN	192	-0.38077	-1.59566	0.00033	0.01657	0.01570
HALLMARK_CHOLESTEROL_HOMEOSTASIS	60	-0.44568	-1.56654	0.00909	0.22608	0.21418
HALLMARK_ESTROGEN_RESPONSE_LATE	193	-0.33080	-1.38714	0.01356	0.22608	0.21418

GSEA: Gene Set Enrichment Analysis
